# Cerebral blood flow characteristics of drug-naïve attention-deficit/hyperactivity disorder with social impairment: Evidence for region–symptom specificity

**DOI:** 10.3389/fnins.2023.1149703

**Published:** 2023-03-21

**Authors:** Kangfuxi Zhang, Jing Yuan, Xuyao Pei, Zhao Fu, Yilu Zhao, Na Hu, Yufeng Wang, Li Yang, Qingjiu Cao

**Affiliations:** Peking University Sixth Hospital, Peking University Institute of Mental Health, NHC Key Laboratory of Mental Health (Peking University), National Clinical Research Centre for Mental Disorders (Peking University Sixth Hospital), Beijing, China

**Keywords:** attention-deficit/hyperactivity disorder (ADHD), social impairment, cerebral blood flow (CBF), arterial spin label, comorbid

## Abstract

**Background:**

Social deficits are among the most important functional impairments in attention-deficit/hyperactivity disorder (ADHD). However, the relationship between social impairment and ADHD core symptoms as well as the underlying cerebral blood flow (CBF) characteristics remain unclear.

**Methods:**

A total of 62 ADHD subjects with social deficits (ADHD + SD), 100 ADHD subjects without social deficits (ADHD-SD) and 81 age-matched typically developing controls (TDC) were enrolled. We first examined the correlation between the Social Responsiveness Scale (SRS-1) and ADHD core symptoms (inattention, hyperactivity, and impulsion) and then explored categorical and dimensional ADHD-related regional CBF by arterial spin labeling (ASL). For the categorical analysis, a voxel-based comparison of CBF maps between the ADHD + SD, ADHD-SD, and TDC groups was performed. For the dimensional analysis, the whole-brain voxel-wise correlation between CBF and ADHD symptoms (inattention, hyperactivity/impulsivity, and total scores) was evaluated in three groups. Finally, correlations between the SRS-1 and ADHD-related regional CBF were investigated. We applied Gaussian random field (GRF) for the correction of multiple comparisons in imaging results (voxel-level *P* < 0.01, and cluster-level *P* < 0.05).

**Results:**

The clinical characteristics analysis showed that social deficits positively correlated with ADHD core symptoms, especially in social communication and autistic mannerisms domains. In the categorical analysis, we found that CBF in the left middle/inferior temporal gyrus in ADHD groups was higher than TDCs and was negatively correlated with the social motivation scores. Moreover, in dimensional analysis, we found that CBF in the left middle frontal gyrus was negatively correlated with the inattention scores, SRS total scores and autistic mannerisms scores in ADHD + SD subjects.

**Conclusion:**

The present study shows that inattention, hyperactivity, and impulsivity may be responsible for the occurrence of social deficits in ADHD, with autistic traits being another significant contributing factor. Additionally, CBF in the left middle/inferior temporal gyrus and the left middle frontal gyrus might represent the corresponding physiological mechanisms underlying social deficits in ADHD.

## 1. Introduction

Attention-deficit/hyperactivity disorder (ADHD) is a common childhood-onset neurodevelopmental disorder characterized by age-inappropriate inattention, hyperactivity, and impulsivity (Vahia, 2013). It is often first identified in school-aged children when it negatively affects school achievement and family, peer, and social interactions (Posner et al., 2020). Social deficits are among the most important functional impairments in ADHD, affecting approximately 52–82% of ADHD patients (Hoza, 2007; Harpin et al., 2016). Children with ADHD frequently display social difficulties such as aggressive social behavior and poor peer regard, and the symptoms always appear from early childhood and continue into adulthood, causing long-term and negative effects in ADHD (Nijmeijer et al., 2008). Specifically, social impairment is one of the predictors of poor long-term prognosis in ADHD, as deficits in social functioning can predict impairments such as school difficulties, risk-taking behavior, and even delinquency (Kofler et al., 2015). Although attention deficit, impulsivity and hyperactivity will improve to a certain extent with growth and development, the social performance of ADHD children will continue to be poor and even worsen, resulting in more adaptation issues (Kofler et al., 2015).

Previous research suggests that social impairment in ADHD subjects may exist in conjunction with and apart from the disorder’s core symptoms (Ray et al., 2017). Inattention symptoms impair their ability to notice social cues and to learn social skills through observation (Zoromski et al., 2015; Humphreys et al., 2016). Hyperactivity and impulsivity symptoms in ADHD are associated with aggressive and overbearing interactions, which lead to peer exclusion and being disliked by peers (Zoromski et al., 2015; Humphreys et al., 2016). In addition, other characteristics are also associated with social impairment in ADHD, such as comorbidity with other disorders (e.g., oppositional defiant disorder and conduct disorder) (Ray et al., 2017) and autistic traits (Mikami et al., 2019). Given that DSM-5 has recognized the comorbidity of ADHD and autism spectrum disorder (ASD), autistic traits are increasingly identified in ADHD (Antshel and Russo, 2019). Evidence suggests that there is significant clinical overlap between ADHD and ASD in terms of impairment in social functioning (Mikami et al., 2019), and children with persistent hyperactive-inattentive symptoms across their lifespan almost entirely display persistently impaired social function (St Pourcain et al., 2011).

Accumulating neuroimaging studies neuroimaging studies have begun to map the social brain (Porcelli et al., 2019; Stoodley and Tsai, 2021). However, the specific neural substrates contributing to social deficits in ADHD remain unclear (Castellanos and Proal, 2012). The medial prefrontal cortex (mPFC), the temporoparietal junction (TPJ), the posterior superior temporal sulcus (pSTS), the inferior frontal gyrus, the anterior cingulate cortex (ACC), and the anterior insula (AI) are the primary regions of the social brain, a network implicated in social function (Porcelli et al., 2019; Stoodley and Tsai, 2021). Structural neuroanatomical and functional abnormalities in many of these social brain regions have been detected in ADHD (Castellanos and Proal, 2012; Mostert et al., 2016; Ball et al., 2019). For example, it has been reported that the gray matter volume is reduced in the frontostriatal, frontolimbic and temporoparietal areas, and the brain regions are hypoactivated during go/no go, response inhibition and attentional tasks in ADHD children and adults (Castellanos and Proal, 2012; Mostert et al., 2016; Ball et al., 2019). Although there is overlap between these abnormalities and the social brain regions, it remains to be seen whether these abnormalities are associated with social impairments in ADHD (Baribeau et al., 2019; Fateh et al., 2022).

Cerebral blood flow (CBF), as a physiological parameter closely related to cerebral metabolism, has received much attention in recent years. The more active and connected neuronal regions of the brain tend to have greater metabolic demands, resulting in increased cerebral perfusion (Vaishnavi et al., 2010; Liao et al., 2013). Arterial spin labeling (ASL) provides a direct, absolute and reproducible measure of regional cerebral blood flow to each voxel of the brain and is widely used to measure cerebral perfusion in the resting brain (Hernandez-Garcia et al., 2019). Compared with functional magnetic resonance imaging (fMRI), ASL is less sensitive to head movements (Hernandez-Garcia et al., 2019), which is important because children with ADHD tend to have greater head movements than typical developmental controls (TDCs) (García Murillo et al., 2015).

The results of previous CBF studies in ADHD have revealed that patients with ADHD are hyperperfused in the left caudate nucleus and in the frontal and parietal regions (O’Gorman et al., 2008; Gonchigsuren et al., 2022). However, some studies have also shown that cerebral blood flow in large resting-state network regions (e.g., ventral attention network, somatomotor network, limbic network) and subcortical regions is significantly lower in the ADHD group than in TDCs (Tan et al., 2020). Due to the heterogeneity of the subjects included in previous studies, there are no consistent conclusions as to whether perfusion in ADHD is elevated or decreased relative to TDCs. Moreover, no studies have been focused on investigating the brain perfusion-symptom relationship in ADHD in the context of social deficit symptoms.

Given that social impairment may underlie the development of ADHD symptoms, investigation of the relationships between social profiles and brain perfusion would deepen our understanding of the pathology of ADHD. In this study, we sought to determine whether the social deficits in ADHD are connected to ADHD characteristics and to investigate the corresponding physiological mechanisms from quantitative and categorical perspectives. We assumed that the core ADHD symptoms, namely, inattention, hyperactivity and impulsivity, might be correlated with social deficits and that there might be characteristic cerebral blood perfusion features that underlie.

## 2. Materials and methods

### 2.1. Participants

A total of 162 medication-naïve children and adolescents with clinically diagnosed ADHD based on the DSM-5 were recruited from the outpatient department of Peking University Sixth Hospital (Beijing, China) from July 2015 to January 2019. Another 81 age-matched TDCs were recruited from schools or nearby communities using recruitment advertisements. The clinical diagnosis was first made by a senior child and adolescent psychiatrist based on the diagnostic criteria of the DSM-5 and was then confirmed by a semistructured interview with the parents and child, performed using the Kiddie Schedule for Affective Disorder and Schizophrenia for School Age Children-Present and Lifetime Version (K-SADS-PL) (Kaufman et al., 1997). Participants in the ADHD group met the following criteria: (1) diagnosis of ADHD according to the K-SADS-PL; (2) age 6-16 years old; (3) Chinese Wechsler Intelligence Scale for Children, third version (C-WISC-III) IQ ≥ 70; and (4) right handed. The exclusion criteria for ADHD participants were as follows: (1) physical illnesses and neurological disorders such as epilepsy; (2) comorbid autism spectrum disorder according to the clinical diagnosis and schizophrenia, affective disorder or other Axis I diagnosed psychiatric disorder according to the K-SADS-PL; (3) history of any psychotic medication use; and (4) not eligible for MRI scanning by virtue of, for example, having metal implants (including non-removable dentures) or suffering from claustrophobia. The participants in the TDC group were governed by the same inclusion and exclusion criteria as the ADHD group except that they did not meet the requirement of any diagnosis of mental disorder according to the K-SADS-PL.

To allow evaluation of the severity of symptoms, the ADHD Rating Scale-IV (ADHD-RS-IV) (DuPaul et al., 1998) and social responsiveness scale (SRS-1) (Constantino, 2002) were completed by the participant’s parent. The SRS-1 was designed to cover the four different aspects of social deficits, e.g., social awareness, social cognition, social communication, and social motivation, in addition to assessing autistic mannerisms. The higher the scores, the worse the social function. We subclassified participants in the ADHD group into two groups, ADHD with social deficits (ADHD + SD) and ADHD without social deficits (ADHD-SD), according to the SRS total scores. Participants with scores≥60 were considered to have social impairments in accordance with previous Chinese studies (Zhou et al., 2017).

### 2.2. Image acquisition

All MRI images were acquired on a GE Discovery 3.0 T MR750 system at the Center for Neuroimaging in Peking University Sixth Hospital. Participants were instructed to relax with their eyes closed, stay still, and think of nothing in particular without falling asleep during the scans. The parameters of the T1 image were as follows: repetition time (TR) = 6.66 ms; echo time (TE) = 2.93 ms; inversion time (TI) = 450 ms; flip angle (FA) = 8°; field of view (FOV) = 256 × 256 mm; matrix = 256 × 256; slice thickness = 1.0 mm with no gap, 180 sagittal slices. Resting-state perfusion imaging was performed using a pseudo-continuous ASL (pcASL) sequence with a 3D fast spin-echo acquisition and background suppression: TR/TE = 4781/11.12 ms; post-label delays = 1525 ms; spiral in readout of 12 arms with 512 sample points; FOV = 220 mm × 220 mm; matrix = 128 × 128; FA = 111°; slice thickness = 3 mm, no gap; 45 axial slices; and the number of excitations = 3.

### 2.3. ASL data processing

The preprocessing of ASL data was performed using the ASLtbx software (Wang et al., 2008) and SPM12 software toolbox.^1^ First, the quantitative CBF images were calculated by using a single-compartment kinetic model because the pCASL and M0 images were acquired separately. Then, the T1 image was coregistered to the perfusion image, which was aligned with the corresponding CBF image. The coregistered T1 image was segmented into gray matter, white matter, and cerebrospinal fluid. Next, the CBF map was normalized into the MNI space based on the transform parameters that were estimated during the segmentation of the T1 image and further resampled to 3 mm × 3 mm × 3 mm. Finally, the normalized CBF images were smoothed with 4 mm FWHM. CBF analysis was conducted within the gray matter mask without the cerebellum (*N* voxel = 45.381). For each participant, the spatially normalized CBF images were transformed to z-scores by subtracting the mean and then dividing by the standard deviation across all voxels in the gray matter mask for further statistical analysis.

### 2.4. Statistical analysis

The demographic and clinical data were compared using SPSS 26.0. For normally distributed variables with homogeneity of variance, ANOVA was used, with the Bonferroni test used for *post-hoc* analysis. A chi-squared test was used to compare the distribution of categorical data between groups. Spearman’s correlation analysis was conducted to examine the correlation between ADHD symptoms and social responsiveness profiles with age and gender as covariates. The significance level was set at *P* < 0.05.

To identify ADHD-related alterations in CBF, we used two sequential approaches: categorical and dimensional analysis. For the categorical analysis, a voxel-based comparison of CBF maps was performed between the ADHD + SD, ADHD-SD, and TDC groups using one-way ANCOVA. For the dimensional analysis, the whole-brain voxel-wise correlation between CBF and ADHD symptoms (inattention, hyperactivity/impulsivity, and total scores) was examined in three groups. For each significant cluster from the above analysis, we calculated the relationship between its CBF and the social profiles. We first extracted the average CBF value of a given cluster and then calculated the partial correlation between each SRS measurement and CBF value in the corresponding groups. All image analyses involved using age, gender and handedness as covariates. For all these imaging results, we applied Gaussian random field (GRF) for the correction of multiple comparisons (voxel-level *P* < 0.01, and cluster-level *P* < 0.05) (Sun et al., 2021).

## 3. Results

### 3.1. Demographic and clinical data

A total of 62 ADHD + SD subjects, 100 ADHD-SD subjects and 81 TDC subjects were included in this analysis (see Table 1). There were no statistically significant differences among the participants in the ADHD + SD, ADHD-SD, and TDC groups in terms of age and handedness (*p* > 0.05). Moreover, there were no differences between the ADHD + SD and ADHD-SD subjects in ADHD-RS-IV scores. However, the three groups did differ in gender (χ^2^ = 9.895, *P* = 0.007) and IQ (*F* = 27.600, *P* < 0.001); the percentage of females and the IQ in the TDC children were higher than those in the ADHD + SD and ADHD-SD children, but there were no statistically significant differences between the ADHD + SD and ADHD-SD subjects.

**TABLE 1:** Demographic and clinical information.

Variables	ADHD + SD	ADHD-SD	TDC	Test stat	*P*	*Post-hoc*
	**(*n* = 62)**	**(*n* = 100)**	**(*n* = 81)**			
Age (month)	122.93 ± 26.21	117.22 ± 23.62	125.58 ± 23.48	*F* = 2.806	0.062	
Gender (male/female)	51/11	77/23	49/32	χ^2^ = 9.895	0.007*	① = ②≠③
Handed (right/mixed right)	53/9	91/9	78/3	χ^2^ = 5.228	0.073	
Full-scale IQ scores	103.04 ± 10.75	104.72 ± 12.93	118.00 ± 16.72	*F* = 27.600	<0.001**	① = ② < ③
**ADHD core symptoms**						
Total	47.88 ± 8.43	46.44 ± 8.13	29.92 ± 6.10	*F* = 136.914	<0.001**	① = ② > ③
Inattention	26.70 ± 3.87	26.39 ± 4.13	16.25 ± 3.73	*F* = 183.905	<0.001**	① = ② > ③
Hyperactivity/impulsivity	21.18 ± 5.73	20.05 ± 5.95	13.67 ± 3.20	*F* = 48.412	<0.001**	① = ② > ③
**Social responsiveness scale**						
Total	73.85 ± 13.28	43.55 ± 10.95	45.78 ± 11.56	*F* = 134.899	<0.001**	① > ② = ③
Social awareness	10.27 ± 2.90	7.41 ± 2.49	8.57 ± 2.53	*F* = 22.672	<0.001**	① > ② = ③
Social cognition	15.79 ± 4.07	10.40 ± 3.52	11.03 ± 3.49	*F* = 42.978	< 0.001**	① > ② = ③
Social communication	23.69 ± 5.92	12.99 ± 5.17	15.30 ± 4.57	*F* = 79.269	<0.001**	① > ② = ③
Social motivation	11.50 ± 3.53	7.26 ± 3.05	6.97 ± 2.93	*F* = 39.298	<0.001**	① > ② = ③
Autistic mannerisms	12.60 ± 4.64	5.49 ± 2.52	3.92 ± 2.80	*F* = 109.482	<0.001**	① > ② = ③

Thirty-seven participants in TDC group have the social responsiveness scale data. ADHD + SD, ADHD with social deficits subjects; ADHD-SD, ADHD without social deficits; TDC, typically developing controls.

*P < 0.05, **P < 0.001.

The correlation between ADHD symptom scores and social impairment symptom scores differed among the subjects in the three groups (see Table 2). In the ADHD + SD group, there was a statistically significant positive correlation between the ADHD-RS-IV total scores and the SRS-1 total scores (*r* = 0.366, *P* = 0.004), as well as the social communication scores (*r* = 0.338, *P* = 0.008) and autistic mannerisms scores (*r* = 0.265, *P* = 0.040). Furthermore, the ADHD-RS-IV IA scores showed a significant positive correlation with the SRS-1 total scores (*r* = 0.279, *P* = 0.031). Similarly, the ADHD-RS-IV HI scores also displayed a significant positive correlation with the SRS-1 total scores (*r* = 0.351, *P* = 0.006), as well as the social communication scores (*r* = 0.333, *P* = 0.009) and autistic mannerisms scores (*r* = 0.275, *P* = 0.033). In the ADHD-SD group, the ADHD-RS-IV total scores were significantly positively correlated with the autistic mannerisms scores (*r* = 0.205, *P* = 0.043), while the ADHD-RS-IV HI scores were significantly negatively correlated with the social motivation scores (*r* = –0.202, *P* = 0.046). The TDC participants demonstrated a closer correlation between ADHD core symptoms and social measures. However, we should take it more conservatively because SRS data was available for only 37 TDCs.

**TABLE 2:** The clinical characteristics between ADHD core symptoms and SRS-1 symptoms.

	Total scores	Social awareness	Social cognition	Social communication	Social motivation	Autistic mannerisms
**ADHD + SD**						
ADHD	0.366**	0.138	0.206	0.338**	0.117	0.265*
IA	0.279*	-0.038	0.218	0.244	0.197	0.172
HI	0.351**	0.231	0.154	0.333**	0.037	0.275*
**ADHD-SD**						
ADHD	0.055	0.172	-0.062	0.065	-0.158	0.205*
IA	0.144	0.113	0.068	0.132	-0.026	0.172
HI	-0.026	0.159	-0.135	-0.004	-0.202*	0.163
**TDC**						
ADHD	0.441**	0.192	0.411*	0.384*	0.290	0.225
IA	0.278	0.105	0.342*	0.247	0.138	0.076
HI	0.506**	0.236	0.385*	0.435**	0.378*	0.327

ADHD + SD, ADHD with social deficits subjects; ADHD-SD, ADHD without social deficits; TDC, typically developing controls; IA, inattention; HI, hyperactivity and impulsivity.

*P < 0.05, **P < 0.01.

### 3.2. ADHD-related regional cerebral blood flow

**FIGURE 1:**
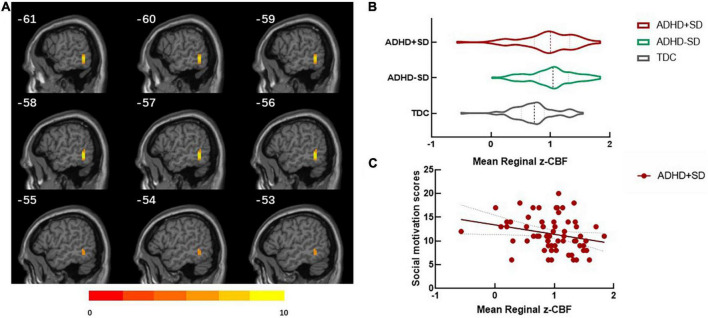
ADHD-related regional cerebral blood flow in the categorical analysis. **(A)** Differences in regional cerebral blood flow (rCBF) between ADHD with social deficit subjects (ADHD + SD), ADHD without social deficit subjects (ADHD-SD), and typically developing controls (TDC). **(B)** The mean rCBF in the left middle/inferior temporal (cluster = 32, and peak MNI = –57, 51, 6) in the three groups. **(C)** The negative correlation between the mean rCBF in the left middle/inferior temporal gyrus and social motivation scores (*r* = —0.275, *P* = 0.034) in the ADHD + SD subjects.

The results of the categorical analysis revealed that, in comparison to the TDC group, both the ADHD + SD and ADHD-SD participants demonstrated a higher CBF in the left middle/inferior temporal gyrus (cluster = 32, and peak MNI = −57, −51, −6). Further *post-hoc* analysis revealed that the ADHD + SD and ADHD-SD participants did not exhibit any significant difference in this particular cluster (see Figure 1).

**FIGURE 2:**
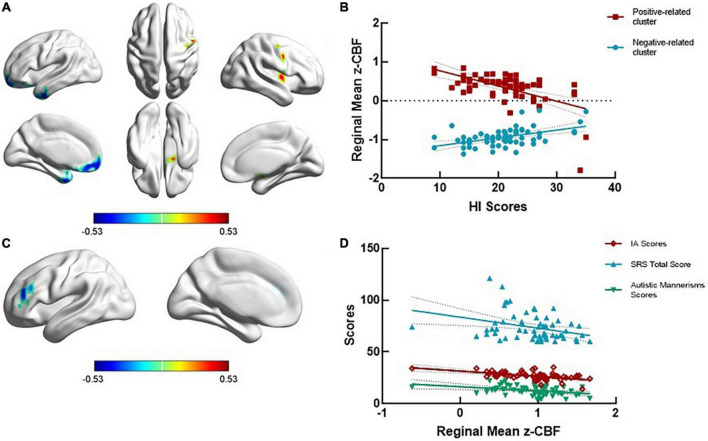
ADHD-related regional cerebral blood flow in the dimensional analysis. **(A,B)** The regional cerebral blood flow (rCBF) was negatively related to ADHD-RS-IV HI scores in the left temporal pole (*r* = —0.528, GRF corrected voxel *P* < 0.01) and positively related to ADHD-RS-IV HI scores in the right precentral gyrus (*r* = 0.549, GRF corrected voxel *P* < 0.01). **(C)** The rCBF value was negatively related to the ADHD-RS-IV IA scores in the left middle frontal gyrus (*r* = —0.521, GRF corrected voxel *P* < 0.01). **(D)** The mean rCBF in the left middle frontal gyrus was negatively correlated with SRS total scores (*r* = —0.321, *P* = 0.012) and autistic mannerisms scores (*r* = —0.358, *P* = 0.005) in the ADHD + SD subjects.

The dimensional analysis showed that in the ADHD + SD group, the CBF value was negatively correlated with the ADHD-RS-IV IA scores in the left middle frontal gyrus (*r* = −0.521, GRF corrected voxel *P* < 0.01), negatively correlated with the ADHD-RS-IV HI scores in the left temporal pole (*r* = −0.528, GRF corrected voxel *P* < 0.01) and positively related to the ADHD-RS-IV HI scores in the right precentral gyrus (*r* = 0.549, GRF corrected voxel *P* < 0.01). In the ADHD-SD group, the CBF value was positively correlated with the ADHD-RS-IV IA scores in the right superior temporal gyrus (*r* = 0.452, GRF corrected voxel *P* < 0.01). No significant correlations were found in the TDC group (see Figure 2).

### 3.3. Correlation between regional cerebral blood flow and social responsiveness scale

The significant cluster in the categorical analysis, the left middle/inferior temporal gyrus, showed that the CBF value was negatively correlated with the social motivation scores (*r* = −0.275, *P* = 0.034) in the ADHD + SD subjects. The partial correlation analysis in the ADHD-SD and TDC subjects was not significant.

The significant cluster in dimensional analysis, the left middle frontal gyrus, was negatively correlated with the SRS total scores (*r* = −0.321, *P* = 0.012) and the autistic mannerisms scores (*r* = −0.358, *P* = 0.005) in the ADHD + SD subjects. No significant correlations were found between the CBF values in other regions and social measures in the ADHD-SD and TDC subjects.

## 4. Discussion

In this study, we found that social deficits in ADHD were related to ADHD core symptoms and ASD traits; moreover, social deficits such as social motivation were associated with ADHD-related brain perfusion. In the clinical characteristics analysis, we found that social deficits were positively correlated with ADHD core symptoms, especially in social communication and autistic mannerisms domains. In the categorical analysis, we found that the CBF in the left middle/inferior temporal gyrus was different between the ADHD and TDC subjects and was negatively correlated with the social motivation scores. Moreover, in dimensional analysis, we found that the CBF in the left middle frontal gyrus was negatively correlated with ADHD-RS-IV IA scores and was negatively correlated with SRS total scores and autistic mannerism scores. Our findings support the hypothesis that the social deficits in ADHD are directly related to ADHD core symptoms, especially impulsivity and hyperactivity. However, non-core ADHD symptoms, such as autistic traits, were also associated with social deficits. Although no precise mechanism by which ADHD children suffer from social deficits is known, the present study was focused on ADHD and social deficit symptoms, as well as the CBF of the corresponding brain region in the hope that such research would promote the discovery of the mechanisms underlying ADHD with social deficits.

The positive correlation between social deficits and ADHD core symptoms in our study indicated that the more severe the attention deficit, impulsivity and hyperactivity, the more severe the social impairments. With regard to social deficit dimensions that are closely related to ADHD core symptoms, social communication and autistic mannerism domains showed a significant positive relationship with ADHD-RS-IV total scores and HI scores. Social communication refers to the ability to respond to social cues (e.g., expression) and is the “motor” aspect of social behavior (Constantino et al., 2004). There is evidence that the core symptoms of ADHD are associated with elevated levels of social impairments (Zoromski et al., 2015; Humphreys et al., 2016; Ray et al., 2017). Hyperactivity and impulsivity lead to disruptive, negative behaviors such as bossiness, aggression, and acting without thinking in peer situations, which are off-putting to peers, resulting in children with ADHD being highly peer-rejected (Humphreys et al., 2016). Due to inattention, individuals with ADHD may ignore social cues and fail to engage in interactive behaviors (such as listening, giving, and receiving), which are critical to successful social interactions. Inattention and disengagement increase peer alienation, further limiting the social interactions needed to gain adequate social knowledge (Zoromski et al., 2015).

The correlation between ADHD core symptoms and ASD traits suggested that the ASD characteristics of ADHD may be another important reason why ADHD children are prone to having social deficits (Mikami et al., 2019), as 15–25% of youth with primary diagnoses of ADHD demonstrate ASD symptoms (Grzadzinski et al., 2016). ADHD and ASD are both neurodevelopmental disorders that begin in childhood, and they are often comorbid and share overlapping symptoms (Mayes et al., 2012; Thapar et al., 2017). Children with each condition show transdiagnostic impairments in social behavior, social cognition, and peer regard. There are both commonalities and distinctions in the manifestation of social problems between ADHD and ASD (Bora and Pantelis, 2016; Mikami et al., 2019). Genetic evidence suggests that the two disorders share a common genetic mechanism, especially in domains such as inattention and communication difficulties (Baranova et al., 2022).

In our categorical analysis, we found that the CBF in the left middle/inferior temporal gyrus had no significant correlation with ADHD symptoms, but the CBF in this region was significantly higher in the ADHD subjects. This is consistent with previous studies, which have confirmed increased activity in the left middle/inferior temporal gyrus in ADHD subjects by fMRI (Hong et al., 2017; Arrington et al., 2019). Moreover, we found that this region was significantly associated with social motivation deficits in ADHD and that the lower the perfusion was, the worse the social motivation impairment. Social motivation refers to the interest in adopting social interpersonal behavior and starting social interactions with others (Clements et al., 2018). As part of the pSTS, the left middle/inferior temporal gyrus is involved in both mirroring and mentalizing brain networks and is activated during behavioral observation and understanding of behavioral intentions (Zilbovicius et al., 2006), which is important in social motivation (Porcelli et al., 2019). A possible explanation for the significantly higher CBF in the ADHD subjects than in the TDC subjects was that the brain compensates for the dysfunction by improving local brain activity, and social motivation function was better compensated in the ADHD-SD group; however, in the ADHD + SD group, this function was dysregulated. Previous neuroimaging studies have reported that pSTS hyperactivity may compensate for social deficits associated with amygdala dysfunction in individuals with higher autistic traits (Xu et al., 2022). However, whether this compensatory mechanism is also present in ADHD needs further confirmation.

Based on our dimensional analysis, we found that social deficits were associated with IA-related regions and that the lower the CBF in the left middle frontal gyrus, the more severe the attention deficit, social impairments and autistic behavior. The involvement of the left middle frontal gyrus in both IA and social deficits is not surprising given that prior studies have implicated this region as a part of the multiple demand system (Koyama et al., 2017). The findings confirmed the insights that autistic characteristics may lead to social deficit predisposition in ADHD. A number of studies have elucidated the role of the left middle frontal gyrus in social function, especially in processing social information and social perception (Birgit et al., 2006). Moreover, it is well documented that the left middle frontal gyrus is abnormal in ASD and correlates with dysfunctions such as face emotion processing deficits (Kryza-Lacombe et al., 2020) and impaired perception of complex sounds (Boddaert et al., 2003). Moreover, the middle frontal gyrus is part of the dopamine-rich frontostriatal circuits (Cheon et al., 2003) and is involved in attention processing; specifically, the right side is associated with sustained attention, and the left side is associated with selective attention (Song et al., 2019). Consistent with our study, previous studies have reported that deficits in processing speed and attention in ADHD were associated with a thinner cortex (Narr et al., 2009), lower activation (Loo et al., 2004; Weber et al., 2007), and poorer metabolism (Tafazoli et al., 2013) in the left middle frontal gyrus. To date, only a few brain imaging studies have directed at social deficits in ADHD, and their findings are consistent with our findings; namely, impaired dynamic and static functional connectivity values in the left middle frontal gyrus have been implicated in social dysfunction in ADHD (Chen et al., 2020; Fateh et al., 2022). Collectively, the previous and current findings indicate the importance of the left middle frontal gyrus in attentional and social function, and improving the CBF in this region may improve these functions.

## 5. Limitations

The study has several limitations. First, the SRS was designed to be a social functioning measure scale for ASD, which may explain why the symptoms in ADHD are closely related to autistic traits in our findings. Moreover, there were only 37 cases with SRS scale results in the TDC group, which may have resulted in statistical false positives. We need to be more conservative about the relationship between ADHD and social deficit symptoms in the TDCs group.

## 6. Conclusion

In summary, by interpreting our results, we demonstrated the relationships between core ADHD symptoms and social deficit symptoms and found that inattention, hyperactivity, and impulsivity may be responsible for the occurrence of social deficits in ADHD. In particular, we found that social deficits in ADHD may not entirely result from ADHD symptoms alone, and the existence of autistic traits was another significant contributing actor. Moreover, CBF in the left middle/inferior temporal gyrus was related to social motivation, and CBF in the left middle frontal gyrus was related to the SRS total scores and autistic mannerisms, which might represent the corresponding physiological mechanisms underlying the social deficits in ADHD.

## Data availability statement

The raw data supporting the conclusions of this article will be made available by the authors, without undue reservation.

## Ethics statement

The studies involving human participants were reviewed and approved by the Medical Ethics Committee of Peking University Sixth Hospital. Written informed consent to participate in this study was provided by the participants’ legal guardian/next of kin. Written informed consent was obtained from the individual(s) and minor(s)’ legal guardian/next of kin, for the publication of any potentially identifiable images or data included in this article.

## Author contributions

KZ analyzed the data and finished the original manuscript draft. JY and XP collected the data. NH scanned the MRI. ZF and YZ organized the clinical information. QC and LY reviewed the methods and the whole manuscript. YW supervised the project. All authors contributed to the article and approved the submitted version.

## Footnotes

1
http://www.fil.ion.ucl.ac.uk/spm
